# Reinforced Chitosan Active Film with Deep Eutectic Solvent and Chestnut Shell Extract: Physicochemical Characteristics, Structure, and Application in Strawberry Preservation

**DOI:** 10.3390/foods15183217

**Published:** 2026-09-11

**Authors:** Jialin Xue, Yating Sun, Meng Wang, Lixiang Huai, Yuting Xiao, Zhenyin Lu, Zhongxu Li, Xianjin Zhou, Haoran Wang, Ruiguo Cui, Lijun Song

**Affiliations:** 1 College of Food Science and Technology, Hebei Normal University of Science and Technology, Qinhuangdao 066600, China; 15690269842@163.com (J.X.); 15232168898@163.com (Y.S.); 15531701889@163.com (L.H.); 19931843390@163.com (Y.X.); 19558926261@163.com (Z.L.); 17332327621@163.com (Z.L.); rannar1126@163.com (H.W.); ruigc_hnust@163.com (R.C.); 2 Chestnut Research Center, Hebei Normal University of Science and Technology, Qinhuangdao 066604, China; 13223362010@163.com; 3 Bazhoujiamu Agroscience Co., Ltd., Korla 841000, China; 19511131635@163.com; 4 Hebei Key Laboratory of Natural Products Activity Components and Function, Hebei Normal University of Science and Technology, Qinhuangdao 066004, China; 5 Hebei Fruit Processing Technology Innovation Center, Hebei Normal University of Science and Technology, Qinhuangdao 066600, China

**Keywords:** chestnut shell extract, deep eutectic solvent, active packaging film, physicochemical characteristics, strawberry preservation

## Abstract

In this study, ternary composite active packaging films composed of chitosan (CH), deep eutectic solvent (DES), and chestnut shell extract (CSE) were prepared. The physicochemical properties, structural characteristics, and application in strawberry preservation were studied. The results show that the DES hydrogen-bond network may promote interactions between CSE and CH, enhancing compatibility and stability. At 5% CSE addition, the maximum values were observed for antioxidant activity (DPPH: 80.49%, ABTS: 86.69%) and antibacterial activity (Antibacterial Zone Diameter (AZD): 27.61 mm for *Escherichia coli E. coli* and 27.15 mm for *Staphylococcus aureus*). FTIR analysis indicated the hydrogen bonding between CSE and the polymer matrix. XRD results indicated that CSE addition enhanced the crystallinity of the film matrices, while SEM revealed that it promoted the formation of a dense structure. Molecular docking results provide computational evidence suggesting that DES may serve as an interfacial bridge connecting CH and CSE. Satisfactorily, after treatment with the CH–DES–CSE-5% film, the shelf life of strawberries was significantly extended. After 8 days of storage, the decay rate (36.67%) was significantly lower than that of the control group (96.67%). The original quality indicators were well maintained. This study provides a theoretical basis for both the valorization of chestnut shell waste and the development of CSE-based active food packaging materials.

## 1. Introduction

Postharvest fruits are susceptible to decay and loss of commercial value due to various factors (temperature, light, oxygen, microorganisms, etc.) [1]. It is reported that 25–50% of fruits harvested worldwide decay annually due to insufficient preservation strategies [2]. This results in resource waste and economic losses. To address this issue, various approaches have been explored, including petroleum-based plastic packaging, physical treatments (ozonation and high-pressure processing), and chemical preservatives (formaldehyde, copper sulfate, potassium sorbate, nitrite, etc.) [3]. These methods can prolong the shelf life of fruits. However, they also have significant limitations, such as complicated procedures, high cost of equipment, and even some harmful additives. These conventional methods can be economically inefficient and pose potential hazards to human health, which are inconsistent with sustainable development. Therefore, the development of safe, biodegradable, and multifunctional green active packaging materials has become an urgent demand for the fruit preservation industry.

In recent years, as an environmentally friendly and biodegradable packaging material, active packaging films using natural biopolymers (such as polysaccharides, proteins, lipids, etc.) as the film-forming matrices have received extensive attention [4]. These matrices crosslink with one another to form a network structure, resulting in active packaging films possessing certain barrier properties [5]. The physicochemical characteristics of active packaging films based on different substrates are different. For example, the lipid-based active packaging films are brittle with poor mechanical properties because of their lack of cohesiveness and structural integrity [6]. Shi et al. (2024) [7] found that protein films have good water vapor and oxygen barrier properties. However, as noted by Zhu (2021) [8], proteins may pose adverse effects on sensitive individuals. In addition, due to the hydrophilic nature of protein films, the mechanical performance of protein-based films is not ideal after their formation, which restricts the application of protein films in food packaging. In contrast, polysaccharide-based active packaging films exhibit excellent mechanical and gas barrier properties [9]. Chitosan (CH) is a linear high-molecular-weight polysaccharide obtained by the deacetylation of chitin. It has excellent biological activity and exhibits a natural broad-spectrum antibacterial effect [10]. The non-toxicity, antioxidant activity and excellent biodegradability of chitosan made it a good film-forming material [4]. It has been reported that chitosan can be applied as a physical barrier on the surface of fruits and vegetables to reduce the decay rate and extend the storage period of fruits and vegetables [11]. Additionally, the chitosan-based active packaging film can reduce the growth of microorganisms, protect the cell structure, and extend the shelf life of fruits [12]. However, the pure chitosan films cannot meet the multiple functional requirements in the food packaging industry [13]. Liu et al. (2024) [13] and Ren et al. (2022) [14] reported that pure chitosan films usually exhibit relatively poor antioxidant and antibacterial properties, which limits their practical application in active food packaging. Moreover, studies have shown that pure chitosan films also possess relatively weak UV barrier properties [15].

Polyphenolic compounds are often incorporated into film-forming solutions to enhance the antioxidant and antibacterial effects of films [3]. For instance, the freshness of strawberries has been better maintained by incorporating resveratrol into chitosan-based film solutions [10]. The preservation effect of strawberries was prominently elevated by adding hawthorn leaf extract to chitosan-based film [16]. Chestnut shells account for about 10% of the total weight of chestnuts and are often discarded as waste, causing environmental pressure and resource crises [17]. In our prior research, a total of 36 compounds were detected in the chestnut shell extract (CSE), including 28 phenolic compounds (Table S1). CSE exhibits excellent antioxidant properties, and it can alleviate oxidative damage by eliminating free radicals [18]. Given its rich polyphenol composition and antioxidant activity, the extract from chestnut shells is expected to be used as a functional additive in chitosan films. However, at present, there are still relatively few studies on the application of chestnut shell extract in the film-forming system. Notably, the intermolecular interaction between polyphenols and chitosan is relatively weak, and their interfacial compatibility is poor. In the film system, they tend to aggregate and cause phase separation [15]. The enhancement of antioxidant and antibacterial activities is often accompanied by the decline in mechanical strength, structural density and barrier performance. This limits the industrial application of polysaccharide–polyphenol composite films.

In order to further improve the protective effect of the active packaging film, adding plasticizers proves an efficient strategy to boost the mechanical strength of CH films [19]. Glycerol and ionic liquids are commonly used as plasticizers. However, its effect is limited to weakening intermolecular forces and enhancing segmental mobility, while failing to improve the interfacial compatibility between polyphenols and the matrix. And they inevitably suffer from drawbacks such as potential toxicity and poor cost-effectiveness [20]. Thus, developing a substitute for conventional plasticizers has become imperative. Deep eutectic solvents (DESs) have recently attracted widespread attention because they are environmentally benign, inexpensive, high solubility for polysaccharides, biodegradable, and effective plasticizers [21]. DESs are formed by combining a hydrogen bond acceptor (HBA) and a hydrogen bond donor (HBD) through hydrogen bonding interactions [18]. Choline chloride (ChCl) is an inexpensive, biodegradable, and non-toxic quaternary ammonium salt commonly employed as an HBA [21]. Previous studies have confirmed that choline-chloride-based DES can be used as an efficient plasticizer to significantly improve the mechanical properties of chitosan films [22]. Chitosan films with a choline chloride–lactic acid DES plasticizer displayed significantly enhanced water vapor transmission rate (WVTR), solubility, and water absorption capacity [23]. Meanwhile, DES composed of ChCl and citric acid exert a significant effect on morphology, mechanical properties, and barrier characteristics of pectin films used in double-lip duck tongues [24]. Moreover, the incorporation of DES can also improve thermal stability, antibacterial properties, and antioxidant activity of CH films [25]. The advantage has also been verified in lignin nanoparticle films [26] and rice starch films [27]. More importantly, DESs can exhibit excellent dissolution and dispersion capabilities for polyphenols through multiple hydrogen bonding interactions [18]. This means that DES possesses the dual functions of both a plasticizing matrix and a dispersing agent for polyphenols. Theoretically, DESs have the potential to serve as a hydrogen-bond bridging medium, which may regulate the mechanical properties of the chitosan matrix and improve the interfacial compatibility between polyphenols and the matrix. Such effects could collectively contribute to the enhanced mechanical performance and biological activity of the composite film. However, current applications of DESs in chitosan films remain largely confined to their role as plasticizers. There are few reports on the uniform dispersion of chestnut shell polyphenols in chitosan membrane systems mediated by DES. Therefore, improving the dispersibility of chestnut shell extract in chitosan matrix with DES is a key approach to achieving high-value resource utilization of chestnut shell waste.

In this study, choline chloride–lactic acid DES was adopted as a potential hydrogen-bond bridging medium to fabricate CH–DES–CSE ternary composite active films. The physicochemical properties, microstructure, as well as antioxidant and antibacterial activities of the as-prepared films were systematically characterized. The potential intermolecular interactions among CH, DES and CSE were preliminarily analyzed based on spectral characterization. Furthermore, the preservation effect of the optimal film was evaluated on postharvest strawberries. The results indicate that the developed active films could delay the quality deterioration of strawberries during storage. The CH–DES-based bioactive films exhibit favorable bioactivity and show promising application potential as multifunctional food packaging materials.

## 2. Materials and Methods

### 2.1. Materials and Reagents

Chestnut (Castanea mollissima cv. “*Yanbao*”) shells were collected in Qinhuangdao city, Hebei province. After collection, the samples were dried naturally, comminuted with a high-speed rotary mill (CS-700; Wuyi Haina Electrical Appliance Co., Ltd., Jinhua, China), and sieved to ≤250 µm (60-mesh). The resultant powder was hermetically sealed and cryo-preserved at −20 °C pending subsequent analyses.

Chitosan (degree of deacetylation ≥ 95%, viscosity: 100–200 mPa·s, molecular weight: 150~200 kDa, CAS No. 9012-76-4), choline chloride, lactic acid, acetic acid, ethanol, sodium hydroxide, methanol, 2,2-diphenyl-1-picrylhydrazyl (DPPH), and 2,2′-azino-bis(3-ethylbenzothiazoline-6-sulfonic acid) (ABTS) were all purchased from Beijing Solarbio Science & Technology Co., Ltd., Beijing, China. All are of analytical purity. *Escherichia coli* SHMCCD25015 (*E. coli*) and *Staphylococcus aureus* ATCC 6538 (*S. aureus*) were purchased from the Shanghai Microbial Culture Collection Center (Shanghai, China). These strains were cultured in Luria–Bertani (LB) broth (Tianjin Huasheng Chemical Reagent Co., Ltd., China) at 37 °C with shaking at 200 rpm for 18–24 h. Fresh “*Hongyan*” strawberries were purchased from the local market, and bean sprout seeds were purchased at the local market in Qinhuangdao.

### 2.2. Preparation of the Films

#### 2.2.1. Preparation of DES

DES was synthesized as previously described [18]. Briefly, choline chloride (HBA) and lactic acid (HBD) were mixed at a molar ratio of 1:2. Then the mixture was transferred to a conical flask and subjected to 80 °C in an aqueous heat bath and then stirred constantly until the solution became fully homogeneous and transparent.

#### 2.2.2. Extraction of Chestnut Shells Extract (CSE)

Following the method described in a previous study [18], 1 g of chestnut shell powder was added to 20 mL of ethanol (50%, *v*/*v*) and extracted using a KQ-500DE ultrasonic extractor (Kunshan Ultrasonic Instrument Co., Ltd., Kunshan, China) assisted extraction at 60 °C, 800 W, and 40 Hz for 45 min. After extraction, the mixture was centrifuged at 5000 rpm for 10 min. Then the supernatant was filtered through 0.45 μm filter membrane and concentrated using a rotary evaporator (N-1100, Shanghai Ailang Instrument Co., Ltd., Shanghai, China). The concentrated solution was freeze-dried to obtain CSE. The compounds in CSE are shown in Table S1.

#### 2.2.3. Preparation of the Films

**Figure 1:**
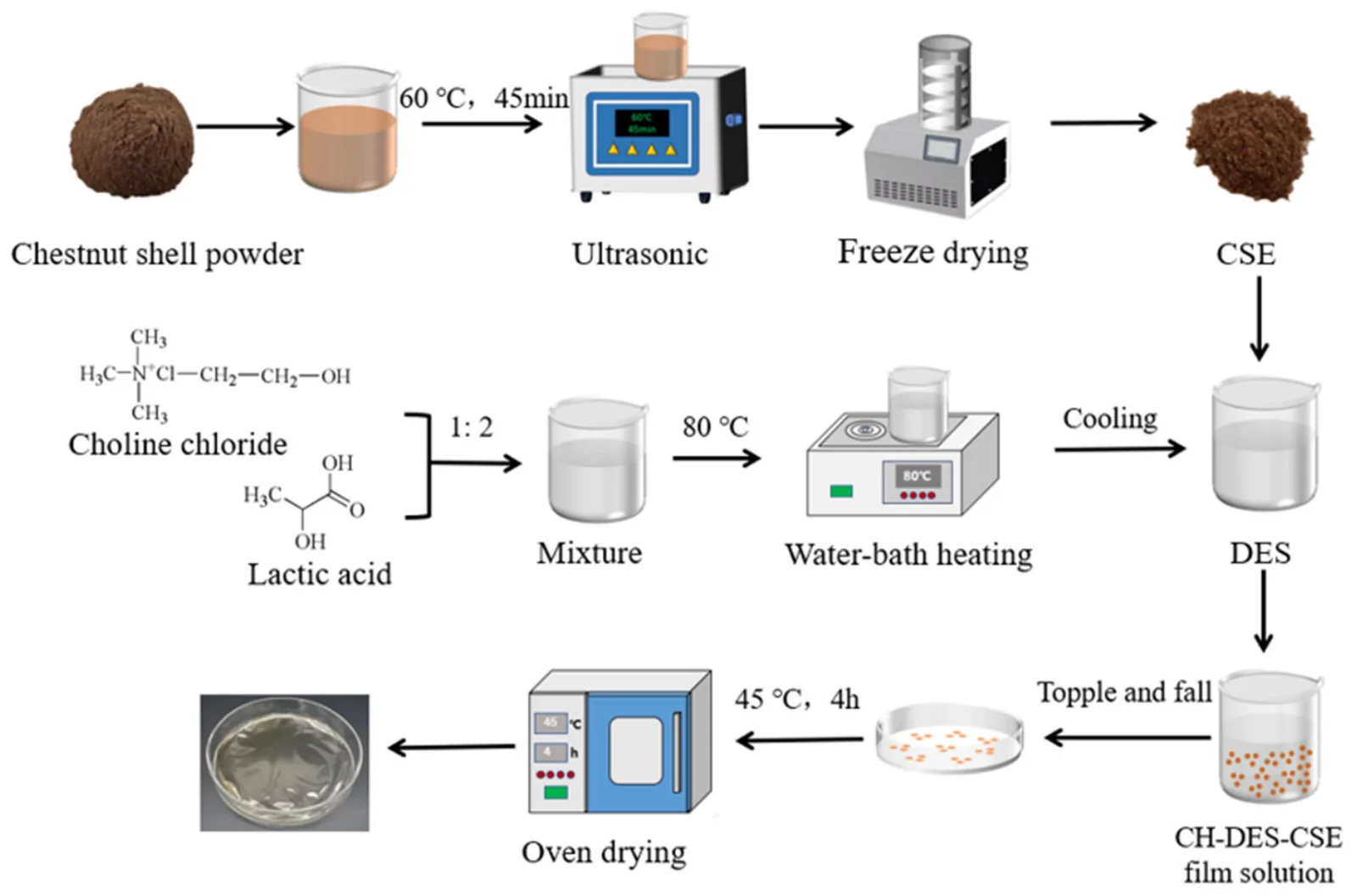
Preparation process of the films.

The preliminary experiments revealed that the physical properties of the pure CH films were poor. Therefore, in this experiment, CH-DES was selected as the control group. The film preparation process is illustrated in Figure 1, which was performed with reference to the method described by Feng et al. (2024) [16] with minor modifications. Briefly, 2.0 g of CH was solubilized in 100 mL dilute acetic acid (1%, *v*/*v*). Then, DES (60 wt% based on the dry weight of CH) and CSE (1, 3, and 5 wt% based on the dry weight of CH) were added to prepare the composite film-forming solutions [21]. A total of 16 mL of the film solution was transferred into a Petri dish (9 cm in diameter) and then dried in a forced-air oven at 45 °C for 4 h to obtain CH–DES–CSE films (1.0%, 3.0%, and 5.0%, *w*/*w*). The films were conditioned at 25 °C and 55 ± 1% relative humidity for 48 h before characterization.

### 2.3. Characteristics of the Films

#### 2.3.1. Color

The color of the films was determined using a colorimeter (YS6003, 3nh Technology Co., Ltd., Shenzhen, China) according to a previous method [18].

(1) $$\Delta E=\sqrt{{(L^{*}-L)}^{2}+{\;(a^{*}-a)}^{2}+{\;(b^{*}-b)}^{2}}$$

where L* represents lightness, a* indicates the red–green axis, b* represents the yellow–blue axis, and ΔE represents the total color difference. Here L, a, and b represent the standard values of the white calibration plate used as the background during film measurements (L = 99, a = 0.04, and b = 0.46).

#### 2.3.2. Optical Properties

The opacity of the film was determined using a UV–visible spectrophotometer (UV-1870, Wujin Instrument Co., Ltd., Changzhou, China) [28]. Opacity was evaluated based on the absorbance measured at 600 nm and calculated using the following formula:

(2) $$\mathrm{Opacity}\;(\%)\;=\frac{A_{600}}{d}$$

where d is the film thickness (mm).

The transmittance of the films was measured with a UV–visible spectrophotometer (UV-1870, Wujin Instrument Co., Ltd., Changzhou, China), and spectra were recorded from 200 to 800 nm.

#### 2.3.3. Mechanical Properties and Thickness

The tensile strength (TS) and elongation at break (EB) of the films were determined using an intelligent electronic tensile tester (XLW, Jinan Languang Electromechanical Technology Co., Ltd., Jinan, China). The samples were cut into strips (15 mm × 50 mm), and the crosshead speed was 200 mm/min. The TS and EB values were calculated employing Formulas (3) and (4).

The thickness of the films was measured using a portable digital micrometer with an accuracy of 0.001 mm, and at least 10 points on each film were measured to obtain an average value.

(3) $$\mathrm{TS}=\frac{F}{x\;\times \;W}$$

(4) $$\mathrm{EB}\;\left(\%\right)=\frac{\Delta L}{L_{0}}$$

where F is the tensile force at break (N), x is the average thickness of the film (mm), W is the width of the film strip (mm), ΔL is the increase in length at break (mm), and L_0_ is the initial gauge length of the film strip (mm).

#### 2.3.4. Moisture Content (MC) and Water Solubility (WS)

The MC was measured as previously described [14]. The film specimens were subjected to drying at 105 °C for 24 h. Then the weight of the sample before and after drying was measured, and the MC was calculated using Equation (5).

*MC* (%) = [(*W*_0_ − *W*_1_)/*W*_0_] × 100% (5)

where W_0_ and W_1_ are the initial weight and the weight after 24 h of drying (g) of sample, respectively.

The WS of the films was determined as previously described [29] with minor modifications. The films were divided into squares (20 mm × 20 mm) and heated at 105 °C for 24 h in an oven until a stable dry weight (W_i_) was reached. Then, the desiccated sample was immersed in distilled water (50 mL) and shaken in an orbital shaker at 25 °C for 24 h. The insoluble material was filtered out, and the sample was dried continuously at 105 °C for 24 h before weighing (Wr). The water solubility of the film was determined employing the following equation:

(6) $$\mathrm{WS}\;\left(\%\right)\;=\;(W_{i}{\;-\;W}_{r})/W_{i}\;\times \;100\%$$

where W_i_ is the initial constant weight of the dried film (g) and W_r_ is the constant weight of the dried insoluble residue (g) after immersion in distilled water and shaking.

#### 2.3.5. Gas Barrier Properties

A gas-transmission analyzer was employed to determine the film’s oxygen transmission rate (OTR) at 25 °C (GTR-10N, Guangzhou Biaoji Packaging Equipment Co., Ltd., Guangzhou, China). Following the infrared procedure in accordance with ASTM F1249 [30], the water vapor transmission rate (WVTR) of the film was recorded employing an infrared water vapor transmission rate tester (model W401L, Guangzhou Biaoji Packaging Equipment Co., Ltd., Guangzhou, China). A film was mounted flat in the test chamber, and the WVTR of the film was measured at 38 °C and 90% relative humidity (RH) [31]. The obtained OTR and WVTR were normalized against sample thickness (d) and pressure difference (ΔP) for the calculation of oxygen permeability (OP) and water vapor permeability (WVP).

(7) $$\mathrm{OP}=\frac{\mathrm{OTR}\;\times \;d}{\Delta P_{1}}$$

where d is the film thickness and ΔP_1_ is the pressure difference across the film.

(8) $$\mathrm{WVP}=\frac{\mathrm{WVTR}\;\times \;d}{\Delta P_{2}}$$

where d is the film thickness and ΔP_2_ is the pressure difference across the film.

#### 2.3.6. Water Contact Angle (WCA)

The WCA of the films was measured following the procedure reported previously [21]. Specifically, a film sample (20 mm × 20 mm) was fixed on a glass slide and subsequently positioned on a contact angle analyzer (Dataphysics OCA20, Filderstadt, Germany). A microsyringe was employed to carefully dispense 1.0 μL of deionized water to the surface of the film. The moment when the water droplet touched the film surface was captured, and the WCA was promptly recorded.

### 2.4. Antioxidant and Antibacterial Activities of the Films

#### 2.4.1. Antioxidant Activity

The DPPH radical scavenging rate was determined according to a previously published method [32]. Briefly, 50 mg of film sample was immersed in 10 mL of 0.2 mmol/L DPPH solution (prepared with 50% *v*/*v* methanol). The mixture was incubated in the dark for 30 min, and the absorbance was recorded at 517 nm.

The ABTS radical scavenging rate was determined as follows: Firstly, the ABTS solution was mixed with an equal volume of potassium persulfate solution (with a certain concentration) and stored overnight [33]. Subsequently, 50 mg of the film sample was added to 10 mL of ABTS solution (2 mmol/L), reacted in the dark for 30 min, and then the absorbance was measured at 734 nm.

(9) $$\mathrm{DPPH}/\mathrm{ABTS}\;\mathrm{radical}\;\mathrm{scavenging}\;\%=(A_{0}-A_{c})/A_{0}\;\times \;100\%$$

where A_0_ is the control absorbance and Ac is the sample absorbance.

#### 2.4.2. Antibacterial Activity

The antibacterial activity was evaluated using *Escherichia coli* (*E. coli*) and *Staphylococcus aureus* (*S. aureus*) [34]. Notably, antibacterial activity was determined for the film-forming solutions rather than the final dried films. Briefly, bacterial suspensions were adjusted to an initial concentration of 1 × 10^6^ CFU/mL. *E. coli* was inoculated on LB agar medium, while *S. aureus* was inoculated on nutrient agar medium. Oxford cups were placed on each inoculated agar plate. Afterwards, 200 μL of the film-forming solution was added into each Oxford cup. Sterile water was used as the control group. All assays were performed in triplicate. The plates were incubated at 37 °C for 48 h. Following incubation, the diameter of the inhibition zone was measured to evaluate antibacterial efficacy [35].

### 2.5. Biosafety Assessment

The biosafety assessment of the films was evaluated using germination experiments of mung bean sprouts [36]. The sprouted seedlings were transferred into a culture vessel with 1% (*w*/*w*) aqueous solution for ongoing cultivation. Plants cultured with tap water were used as controls. The growth status of plants was recorded on 0, 3, 6, and 9 days. The germination rate (%) and mean shoot length (cm) of the mung bean sprouts were also measured.

### 2.6. Structural Characteristics of the Films

#### 2.6.1. Thermogravimetric Analysis (TGA)

The thermogravimetric analyzer (TGA, Netzsch, Selb, Germany) was used to characterize the thermal stability of the films. The tests were conducted within the temperature range of 20–600 °C, with a heating rate of 10 °C/min.

#### 2.6.2. Scanning Electron Microscope (SEM)

SEM of the surface and cross-sections of the films were recorded using a scanning electron microscope (SU8010, Hitachi, Tokyo, Japan). Firstly, the film samples were frozen and fractured in liquid nitrogen. Subsequently, the samples were placed on the sample stage and subjected to gold-sputter-coated treatment to enhance the contrast. The surface and cross-sections of the samples were scanned to observe their microstructure [23].

#### 2.6.3. Fourier Transform Infrared Spectroscopy (FTIR)

The functional groups of film samples were characterized via FTIR according to Liang et al. (2024) [37]. Firstly, the film samples were ground and thoroughly mixed with KBr. After mixing evenly, the mixture was compressed into transparent pellets. Then, the samples were analyzed using Fourier transform infrared spectroscopy (Bruker Tensor 27, Ettlingen, Germany) to obtain the spectra in the range of 4000–400 cm^−1^.

#### 2.6.4. X-Ray Diffraction (XRD)

The crystal structure and crystallinity of film samples were characterized by XRD with an X-ray diffractometer (PANalytical, Almelo, The Netherlands). Measurements were performed from 5° to 50° (2θ) at an accelerating voltage of 40 kV and a scanning rate of 3 °/min. Peak areas and relative crystallinity derived from XRD patterns were calculated using Origin 2021 (OriginLab, Northampton, MA, USA).

### 2.7. Molecular Docking

Two representative and highly abundant polyphenols (calomelanol F and catechin) from CSE were selected for molecular docking simulations. The 3D structures of small molecules were retrieved from the PubChem website and subjected to energy minimization using Discovery Studio 2019. Hydrogens were added using AutoDockTools 4.2, and the receptor and ligand were prepared separately and saved as pdbqt files. Molecular docking was subsequently performed using AutoDock 4.2, and the binding affinities (in kcal/mol) were extracted from the docking outputs. The resultant binding configurations were visualized with PyMOL 3.1 to characterize intermolecular interactions among CH, DES, calomelanol F and catechin.

### 2.8. Preservation Effect on the Strawberry

#### 2.8.1. Application of the Films on the Strawberry

The strawberries were placed in containers and then covered with CH–DES, CH–DES–CSE-1%, CH–DES–CSE-3%, and CH–DES–CSE-5% active composite films. Strawberries without any covering served as the control group. The freshness of strawberries in each covered group and the uncovered group was continuously monitored at 25 °C. The preservation effects of each covered group were observed, and changes in quality indicators of strawberries were studied, including weight loss rate (%), decay rate (%), hardness (N), pH value, and titratable acidity. Measurements were conducted daily, and each measurement was repeated three times.

#### 2.8.2. Weight Loss Rate of Strawberry

Three samples were randomly selected from each group. Their weights were measured using a digital precision scale with an accuracy of 0.01 g. The average of the three measurements was taken as the final weight value. The weight loss rate of strawberry was calculated as the ratio of the cumulative weight loss to the original measured weight, expressed as a percentage [38].

#### 2.8.3. Decay Rate

Strawberries with consistent maturity and quality were randomly divided into 15 groups, with 10 strawberries in each group. Three parallel experiments were conducted for each sample to determine the decay rate of strawberries. Taking an 8-day storage period as a cycle, the strawberries were judged as decayed when they show mold spots [39]. The number of decayed strawberries in each group was recorded daily to calculate the decay rate.

(10) $$\mathrm{Decay}\;\mathrm{rate}\;\left(\%\right)={\;N}_{d}\;/{\;N}_{t}\;\times \;100\%$$

where N_d_ represents the number of decays and N_t_ represents the total number.

#### 2.8.4. Hardness, pH and Titratable Acidity (TA)

The hardness of the sample was determined using a texture analyzer, and 5 strawberries were randomly selected from each group for testing. The pH was determined by measuring the uniformly mixed strawberry homogenate using a pH meter [40]. For the determination of titratable acidity, a 0.1 mol/L NaOH solution was employed, and titration was performed with phenolphthalein as an indicator. The result was expressed as citric acid per 100 g of fresh weight.

### 2.9. Statistical Analysis

All measurements were performed in triplicate and expressed as mean ± standard deviation. Graphs were generated using Origin software (Origin 2021pro, OriginLab Corporation, Germantown, MD, USA). One-way analysis of variance (ANOVA) was conducted using SPSS software (IBM SPSS 27.0), with statistical significance defined at the 5% level (*p* < 0.05).

## 3. Results

### 3.1. Characterization of the Films

#### 3.1.1. Color and Opacity of the Films

**Figure 2:**
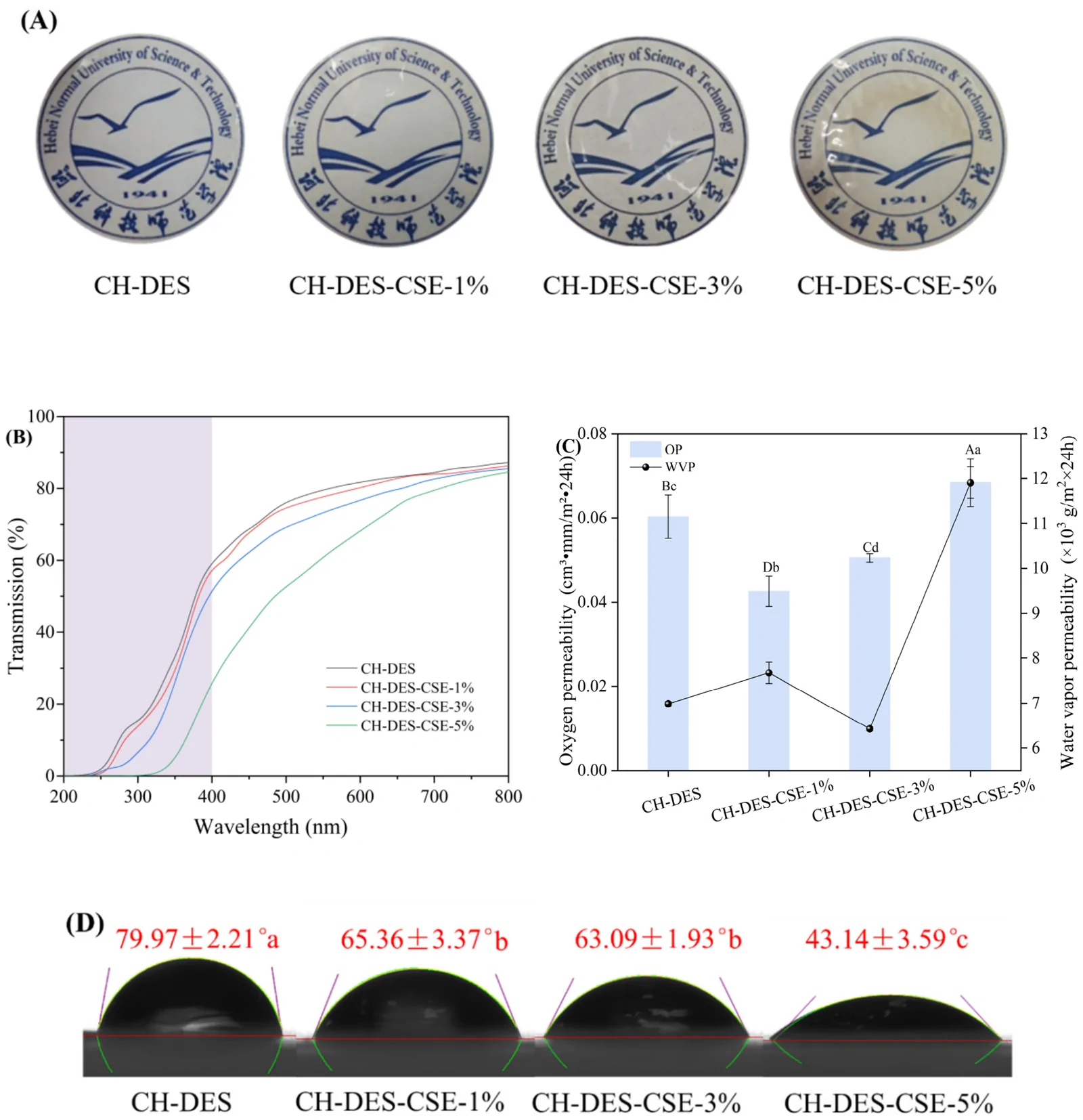
The appearance (**A**), ultraviolet–visible transmission (**B**), oxygen permeability and water vapor permeability (**C**), and water contact angle (**D**) of different films. Different uppercase letters above bars (OP) and lowercase letters beside data points (WVP) in panel C indicate significant differences; different lowercase letters above values in panel D denote significant differences in contact angle among film samples (*p* < 0.05). OP, oxygen permeability; WVP, water vapor permeability.

Figure 2A illustrates the surface morphologies of the films. It can be seen that all the films exhibited smooth surfaces, crack-free morphology, and uniform texture. With the increase in CSE content from 1% to 5%, the color of films changed gradually from light yellow to light reddish-brown. Notably, although the microscopic texture remained homogeneous at 5% CSE addition, visible uneven coloration appeared on the film surface, which was preliminarily attributed to the local aggregation of CSE components. Optical properties of films are summarized in Table 1. As shown in Table 1, with the increase in CSE content, the L* value gradually decreased. Meanwhile, the a*, b*, ΔE, and opacity values showed an increasing trend. These results indicate that higher CSE contents shifted the film color toward the red–yellow region, consistent with the inherent reddish-brown coloration of CSE [41]. The observed variations in color uniformity can be explained as follows. At CSE contents of 1% and 3%, DES facilitated the dispersion of CSE within the chitosan matrix by forming hydrogen bonds with the chitosan chains, thereby enhancing interfacial compatibility, resulting in a uniform and flat composite film. Furthermore, according to the FTIR results, the dispersive effect mediated by hydrogen bonding interactions plateaued as CSE content increased from 3% to 5%. This may be because the hydrogen bonding sites available for DES interaction became saturated. Excess CSE lacking sufficient hydrogen bonding anchoring sites to overcome intermolecular aggregation forces could not be fully stabilized in the membrane solution, leading to molecular aggregation and non-uniform film coloration.

The UV transmission of the films is presented in Figure 2B. As shown in Figure 2B, the UV transmission light transmission of each film gradually increased and reached a higher level near 800 nm. This result suggested that the light transmission ability of the films in the visible and near-infrared bands was enhanced. Compared with CH–DES, the UV transmission was reduced by adding CSE. In particular, the transmission in the range of 200–350 nm decreased sharply to nearly zero when CSE content increased to 5%. This trend was mainly ascribed to the UV transmission absorption effect of polyphenols and flavonoids in CSE. This absorption further enhanced the UV-blocking efficiency of the films [15].

#### 3.1.2. Mechanical Properties

Mechanical properties of films are presented in Table 1. As shown in Table 1, elongation at break (EB) gradually decreased as CSE content increased. Tensile strength (TS) reached a maximum of 22.99 MPa at 3% CSE loading, which was markedly higher than that of the CH–DES control group. At this loading, the increased TS was attributed to the optimal dispersion of CSE mediated by DES and the enhanced intermolecular interactions among CSE, CH, and DES, which together formed a tight internal network. At higher concentrations, excess CSE tended to aggregate within the film matrix and acted as structural defects, leading to a decline in mechanical strength [42]. In addition, the pure CH–DES film exhibited the maximum EB of 85.60%. After CSE incorporation, EB decreased significantly, falling from 85.60% to 55.93% at 3% CSE (a relative decrease of 34.66%). This trend was explained by two reasons. CSE particles filled internal voids of the film matrix, and newly formed hydrogen bonds between CH and CSE restricted chain movement, decreasing matrix flexibility [43]. This performance trend was highly consistent with the evolution of cross-sectional morphology observed in the SEM images and the variations in crystallinity reflected in the XRD patterns.

As shown in Table 1, with the increase in CSE content, the thickness of the film increased and the range was from 0.037 to 0.060 mm. When CSE content was 3% and 5%, the thickness of film increased by 40.54% and 62.16% compared with CH–DES. The increased solid content in films accounted for the thickness variation [20].

#### 3.1.3. Gas Barrier Properties

OTR and WVTR are key indicators for evaluating the ability of thin films to maintain internal gas environments [43]. Figure 2C presents the thickness-normalized oxygen permeability (OP) and water-vapor permeability (WVP) coefficients of the prepared films, which eliminate the influence of variable film thickness and reflect the intrinsic barrier characteristics of the matrix. As illustrated in Figure 2C, CSE addition exerted divergent effects on oxygen and water-vapor barrier performance. The CH–DES–CSE-1% film achieved the lowest OP value, whereas the minimum WVP was observed for the CH–DES–CSE-3% sample. Moderate incorporation of CSE increased film thickness, which lengthens the tortuous diffusion pathways for oxygen and water-vapor molecules. Beyond the physical thickness effect, hydrogen bonding interactions formed between CSE and the amino (-NH_2_) or hydroxyl (-OH) groups of chitosan-strengthened intermolecular forces, reduced polymer free-volume, and retarded gas diffusion, consequently improving the intrinsic barrier performance of composite films [44]. This result was also supported by SEM and XRD analyses. Consistent with earlier reports, OP and WVP are negatively correlated with film crystallinity: higher crystallinity reduces the fraction of amorphous domains, forcing permeating molecules to follow more convoluted diffusion routes and lowering permeability [45].

Nevertheless, further increasing CSE loading to 5% led to a prominent rise in both OP and WVP, even though film thickness continued to increase. This observation demonstrates that adverse structural defects outweighed the barrier benefit brought by greater thickness. At high CSE content, poor dispersion and local aggregation of CSE occurred within the chitosan matrix, deteriorating barrier performance [42]. The elevated WVP of CH–DES–CSE-5% can be partly related to its lower water-contact angle, with hydrophilic domains formed on the film surface facilitating water-molecule adsorption. In addition, the intrinsic hydrophilic nature of choline chloride–lactic acid DES further accelerates water-vapor sorption and transport across the film matrix [46]. As described in the mechanical-property section, CH–DES–CSE-5% showed simultaneous decreases in tensile strength and elongation at break, reflecting impaired structural integrity. The deterioration in barrier performance originates from CSE-driven phase heterogeneity; excess CSE disturbs the continuous compact CH–DES network, generating interfacial voids and agglomerates that act as fast permeation channels for oxygen and water vapor, while also acting as mechanical weak points [47]. Under this defect-dominated condition, the positive contributions from matrix compactness and increased thickness were offset, ultimately resulting in worse barrier properties.

#### 3.1.4. MC, WS and WCA

MC and WS of various films are presented in Table 1. The results revealed a clear downward trend in MC following the addition of CSE. The MC of the CSE groups decreased significantly compared with the CH–DES group, and the rate of decrease was 12.83–15.83%. Nevertheless, no significant difference in MC was observed among the samples of CSE groups. This might be caused by the replacement of water molecules by CSE binding with CH and DES. Alternatively, strong intermolecular interactions between phenolic compounds in CSE and the hydroxyl and amino groups in CH–DES could also account for this. These interactions limited the binding of water molecules to CH–DES [48]. In contrast, WS exhibited an increasing trend with CSE content. This might be caused by the hydrophilic compounds in CSE (e.g., catechins and epicatechins), which disrupted the dense hydrogen bond networks of CH–DES and facilitated water penetration into the matrix [49]. These compounds also could promote water adsorption at the film surface, accelerating the dissolution process [25]. This result was similar to that obtained for the composite film of CSE and chitosan [41].

The WCA of the film can be used to determine its surface properties. A surface with a WCA < 90° is considered hydrophilic, while a surface with a WCA > 90° is considered hydrophobic [19]. The WCA of the films is shown in Figure 2D. From Figure 2D, it can be seen that the WCA of the films, in descending order, was CH–DES (79.97°) > CH–DES–CSE-1% (65.36°) > CH–DES–CSE-3% (63.09°) > CH–DES–CSE-5% (43.14°), which indicates that all films exhibit hydrophilicity. With the increase in CSE content, the WCA decreased. This may be a result of the fact that the hydrophilic nature of DES causes the film to exhibit hydrophilicity as a whole [50]. Additionally, the CSE contains a large number of phenolic hydroxyl groups. With increasing CSE content, the number of hydrophilic sites on the membrane surface and in the bulk also increases [18]. Therefore, the overall hydrophilicity is enhanced.

### 3.2. Antioxidant and Antibacterial Activity

#### 3.2.1. Antioxidant Activity

**Figure 3:**
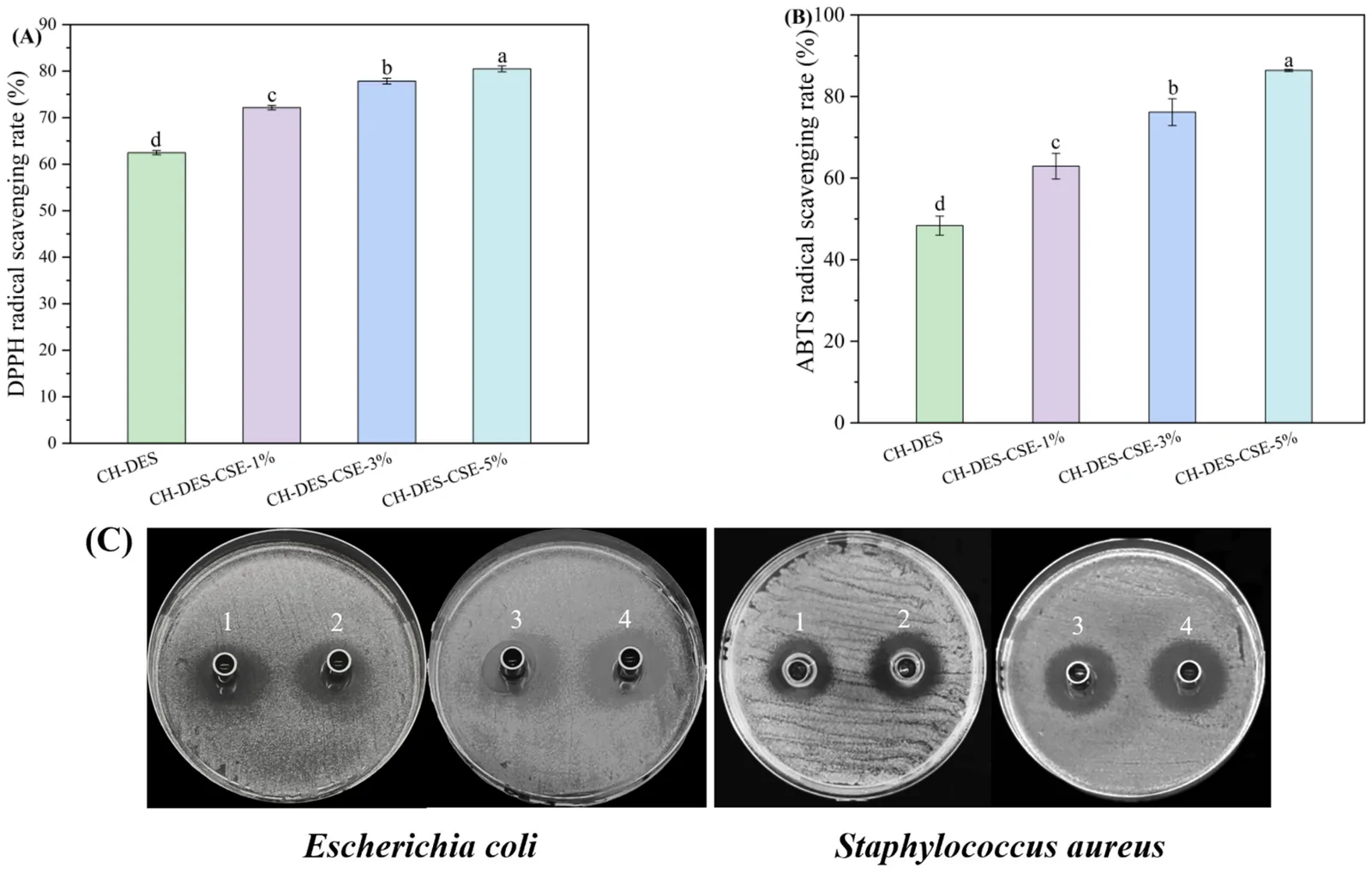
DPPH radical scavenging activity (**A**), ABTS radical scavenging activity (**B**), and antibacterial experiment (**C**) of different films. In Figure 3C, (**1**) CH–DES, (**2**) CH–DES–CSE-1%, (**3**) CH–DES–CSE-3%, (**4**) CH–DES–CSE-5%. Different lowercase letters above bars indicate significant differences among film samples (*p* < 0.05).

The antioxidant activities of the films are presented in Figure 3. As depicted in Figure 3A, the DPPH radical scavenging rate of CH–DES was 62.47%. This might be related to the antioxidant properties of chitosan and DES components [25]. Compared with the control group, the antioxidant activities of the CSE groups were all improved significantly (*p* < 0.05). As the content of CSE increased, the antioxidant activity of the films gradually improved. The DPPH radical scavenging rates of the CSE groups were 72.13% at 1%, 77.84% at 3%, and 80.49% at 5%, respectively. A similar trend was also observed in the ABTS test (Figure 3B). The ABTS free radical scavenging rate for CH–DES was 48.34%, which was 14.57–38.05% lower than those of the CSE groups with different concentrations. This was closely related to the strong antioxidant activity of CSE. Our previous research showed that CSE is rich in polyphenolic compounds, which confer significant antioxidant activity [18]. These antioxidant-enhanced films were beneficial for protecting food from the adverse effects of oxygen and microorganisms [39].

#### 3.2.2. Antibacterial Activity

The antibacterial activity was divided into four levels: ±: 8 mm < antibacterial zone diameter (AZD) ≤ 12 mm; +: 12 mm < AZD ≤ 16 mm; ++: 16 mm < AZD ≤ 20 mm; and +++: AZD > 20 mm [39]. The image of the antibacterial zone (AZ) of the film solutions was shown in Figure 3C. It can be seen that the AZ of the film solution on *E. coli* and *S. aureus* gradually increased with the increase in CSE content.

Table 2 shows the AZD and antibacterial activity intensity of different film solutions. The AZD of *E. coli* ranged from 20.79 to 27.61 mm, and the AZD of *S. aureus* ranged from 21.74 to 27.15 mm. These results indicated that all film solutions exhibited strong antibacterial activity (+++), and this activity was further enhanced with increasing CSE concentration. Yu et al. (2023) [21] indicated that an acidic environment can disrupt the cell membrane, leading to bacterial cell death and thereby exerting an antibacterial effect. The organic acids used in this study have a relatively low pH, which explains the antibacterial effect of CH–DES. Meanwhile, the polyphenolic compounds in CSE are natural components with significant biological activity, showing promising prospects in the field of antibacterial agents. Studies have demonstrated that these polyphenols can effectively inhibit the growth of *E. coli* and *S. aureus* [51]. Given that *E. coli* is a major foodborne pathogen in fruits, this film is expected to effectively inhibit the proliferation of these pathogens, thereby extending the shelf life [7,9]. Together with the barrier properties discussed in the gas barrier properties section, this excellent antibacterial activity provides essential support for reducing decay during subsequent strawberry storage.

In this study only two bacterial strains, *E. coli* (Gram-negative) and *S. aureus* (Gram-positive), were included in the in vitro antibacterial assay. These results only reflect the antibacterial activity of the composite films and are insufficient to fully explain the preservation effect on strawberries because mold and yeast are likely to be the main microorganisms involved in strawberry decay. Future research will further evaluate the antifungal performance of CSE–DES composite films against prevalent molds that cause strawberry decay of strawberries and monitor the dynamic changes in yeast and mold populations during storage, so as to more comprehensively reveal the preservation mechanism and application potential of the films.

### 3.3. Biosafety Assessment

**Figure 4:**
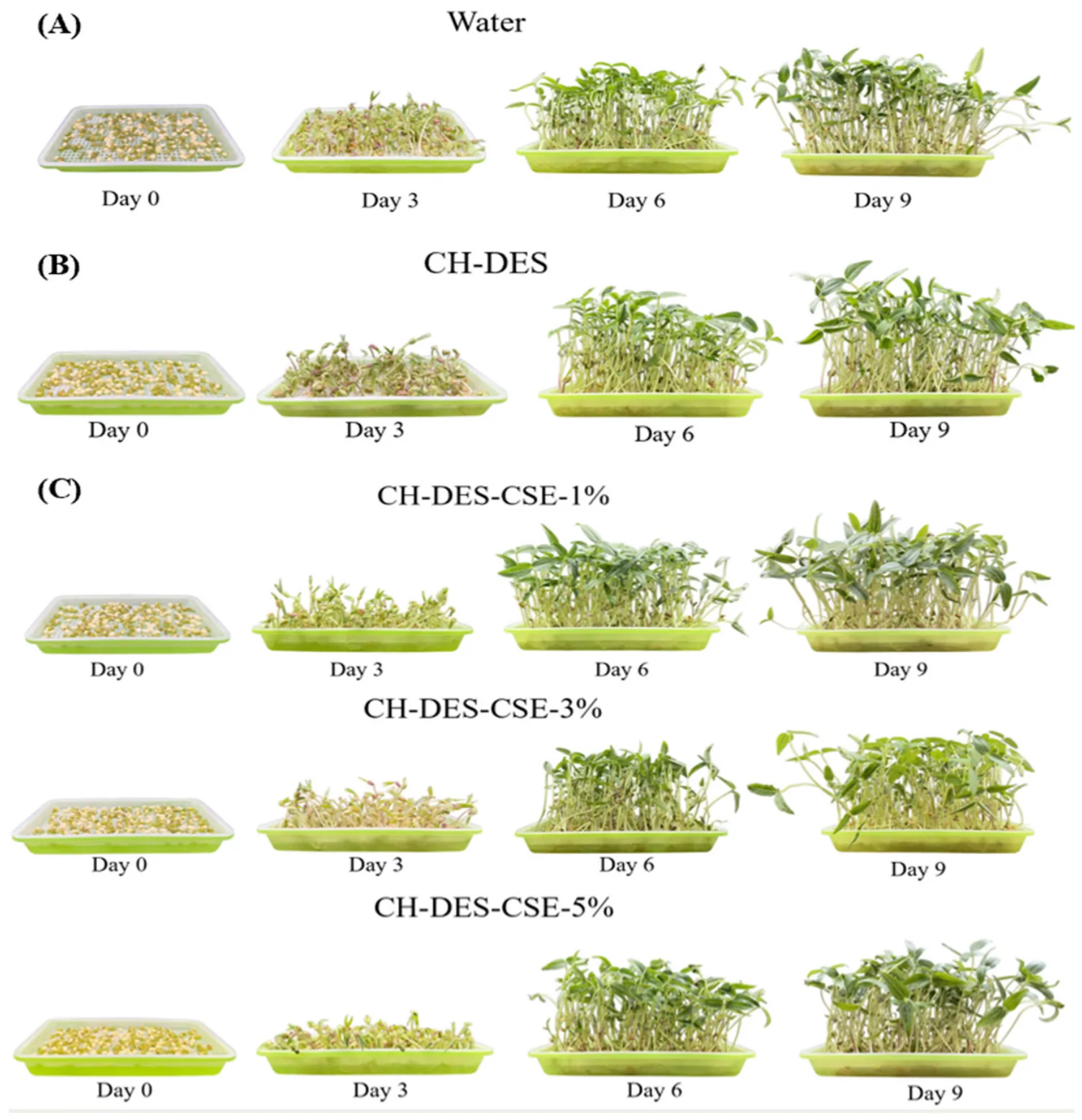
Growth of bean sprout seedlings treated with water (**A**), CH–DES (**B**), CH–DES–CSE-1% (**C**) (**top**), CH–DES–CSE-3% (**C**) (**middle**), and CH–DES–CSE-5% (**C**) (**bottom**) for 9 days.

The germination experiments of mung bean sprouts have been confirmed to be an effective method to evaluate the safety of films [28]. Figure 4 shows the growth status of mung bean sprouts cultivated with tap water and different film solutions (1.0%, *w*/*w*) with various CSE concentrations (0%, 1%, 3%, and 5%) at room temperature. It can be observed that there was no obvious difference in the condition and color of the bean sprouts among all groups.

**Figure 5:**
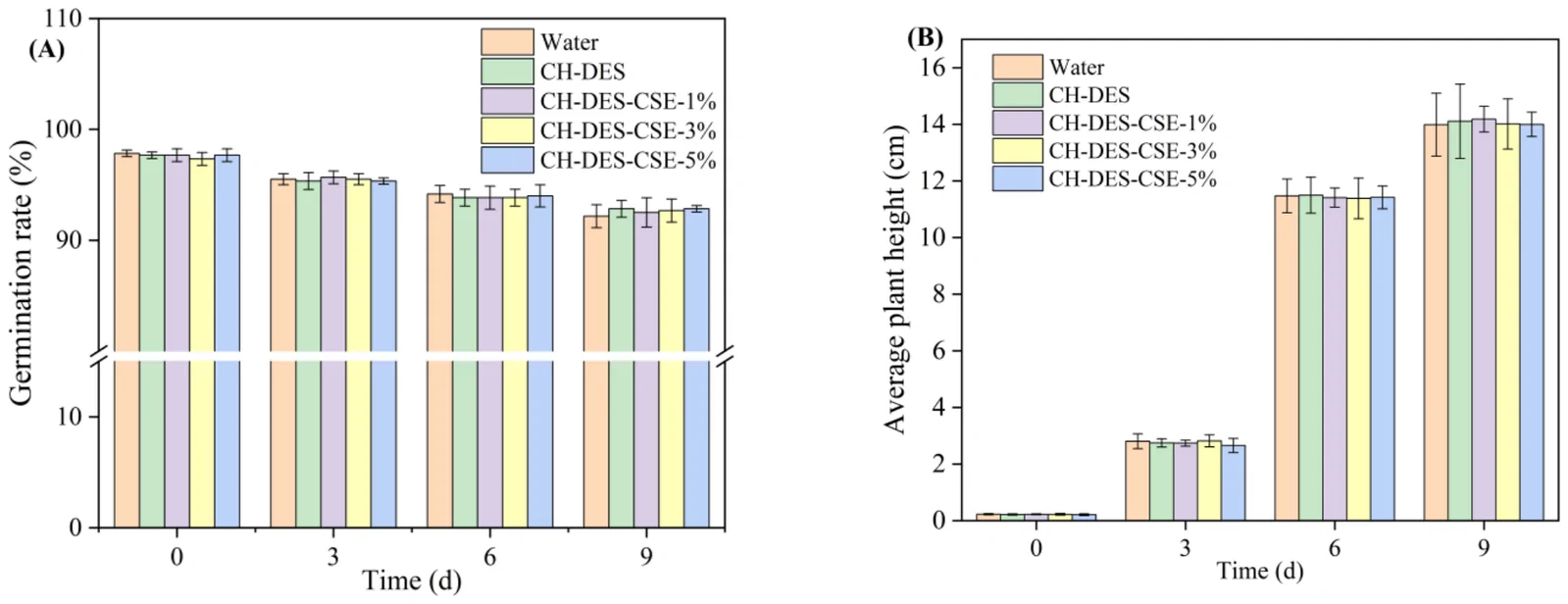
The growth parameters of bean sprout seedlings treated with water and different film solutions for 9 days. (**A**) Germination rate; (**B**) average plant height (values without letter labels showed no significant difference at the *p* < 0.05 level).

The seed germination, as a critical stage in the growth cycle of seedlings, affects the quality of seedlings [52]. Figure 4A–C show the appearance of mung bean sprouts after 9 days of incubation. As shown in Figure 5A,B, the average germination rates of mung bean sprouts irrigated with tap water and four different film solutions were all higher than 92%. Additionally, there was no significant difference in the average height of mung bean sprouts among all groups (*p* > 0.05). These values ranged from 13.97 to 14.18 cm (Figure 5B). These results indicate that the CH–DES and CH–DES–CSE film solutions showed no obvious phytotoxicity toward mung beans under the tested conditions.

The mung bean germination experiment demonstrated that the as-prepared films showed no obvious phytotoxicity under the test conditions. However, this phytotoxicity assessment cannot confirm food-contact biosafety. Further comprehensive safety evaluations are required before practical food packaging applications.

### 3.4. Structural Characteristics

#### 3.4.1. TGA

**Figure 6:**
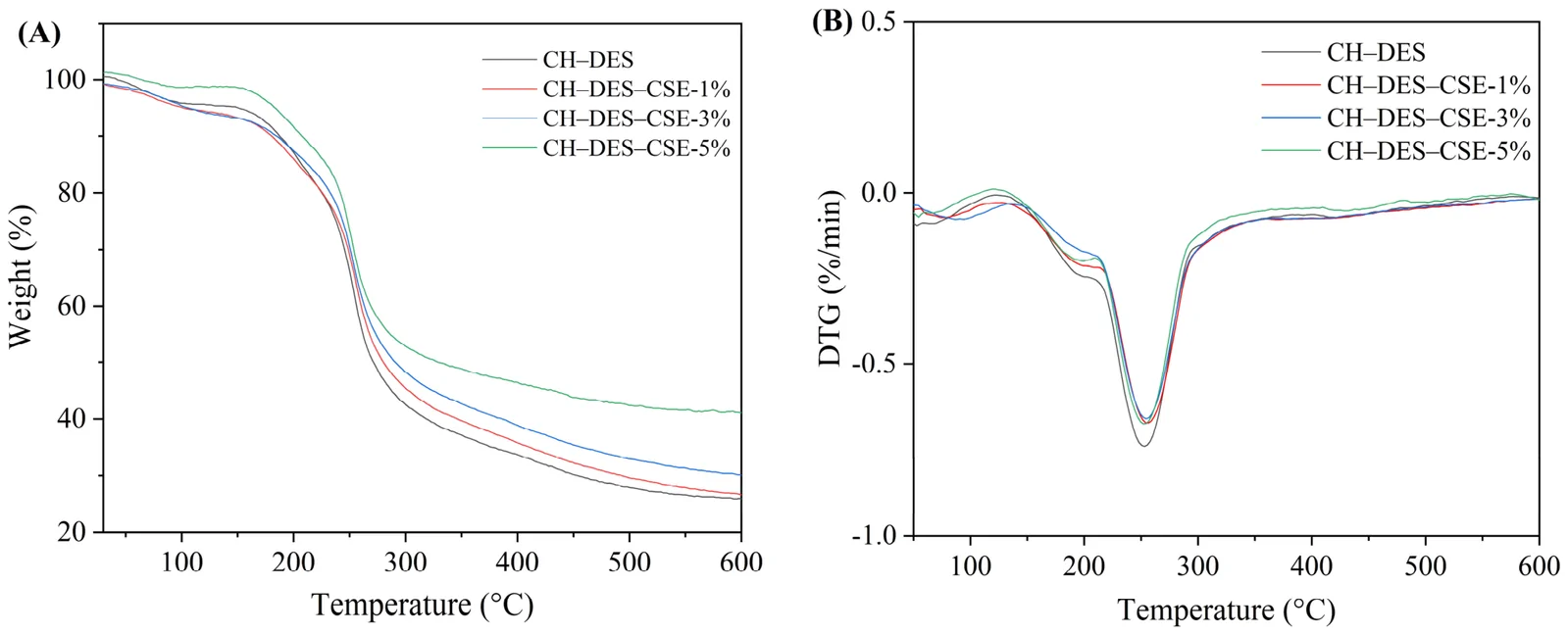
TGA (**A**) and DTG (**B**) of different films.

Figure 6A,B show the TGA and DTG curves of all films, respectively. The TGA curves of all chitosan films exhibited three main weight loss stages in the range of 20–600 °C. The initial stage (20–150 °C) of weight loss was attributed to the evaporation of adsorbed water [53]. The second stage (150–250 °C) showed a significant weight loss corresponding to the thermal decomposition of DES. In the third stage (250–600 °C), the weight loss may be related to the decomposition of CH. Notably the composite film loaded with CSE showed higher thermal stability than the CH–DES film. At 600 °C, the residual weight of CH–DES–CSE-5% was 41% of the initial weight, significantly higher than that of the CH–DES film (26%) (Figure 6A). Furthermore, the DTG curve indicates that the peak degradation temperature of the film increased from 253 °C to 257 °C upon the addition of CSE (Figure 6B). The introduction of CSE was a key factor in enhancing the thermal stability of the system. As the content of CSE increased, the thermal degradation rate of the film decreased significantly, indicating that CSE could effectively inhibit the thermal decomposition process. This enhancement in thermal stability can be attributed to the formation of a more stable intermolecular interaction network among CSE, CH, and DES, as well as the reorganization of the hydrogen bond structure in the CH–DES system [54]. The gradual improvement in thermal stability offers a thermodynamic basis for enhanced system compatibility, which could be associated with the potential bridging effect of DES. This observation is consistent with the optimized mechanical and barrier properties described in Section 3.1.

#### 3.4.2. SEM

**Figure 7:**
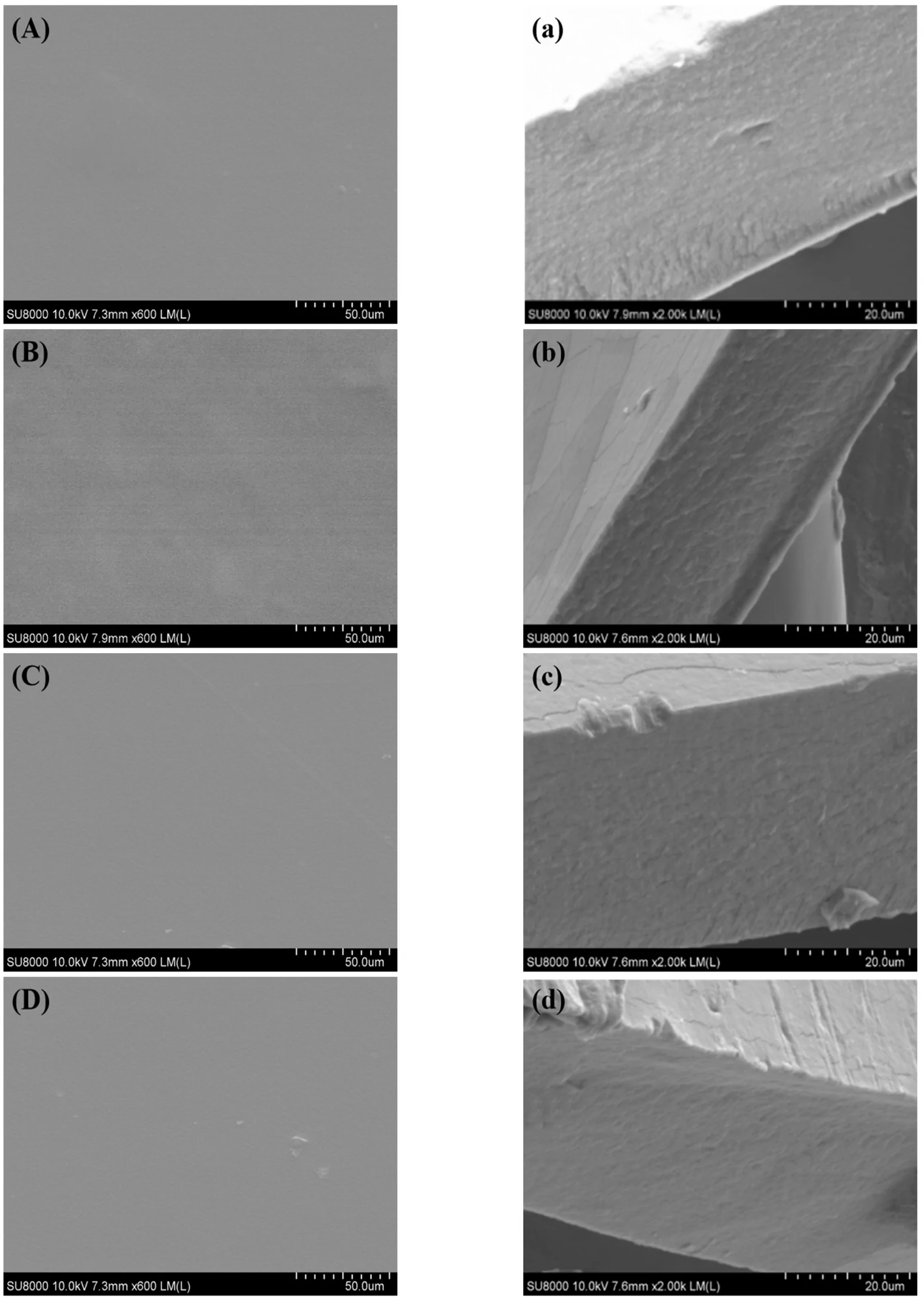
SEM images of different films ((**A**) CH–DES; (**B**) CH–DES–CSE-1%; (**C**) CH–DES–CSE-3%; (**D**) CH–DES–CSE-5%) The corresponding cross-sections of cryo-fractured different films ((**a**) CH–DES; (**b**) CH–DES–CSE-1%; (**c**) CH–DES–CSE-3%; (**d**) CH–DES–CSE-5%).

Figure 7 shows the microstructure of different films. It can be seen that the surface of CH–DES was smooth, uniform, and complete (Figure 7A). Following the CSE addition, no appreciable alterations were detected on the surface.

The cross-sectional morphology showed a distinct pattern of change. The cross-section of the CH–DES control group had obvious pores and a relatively loose structure (Figure 7a). As the CSE addition increased from 1% to 3%, the cross-sectional pores gradually decreased and the structure became increasingly dense and smooth, with no obvious phase separation interface (Figure 7b,c). When the CSE addition reached 5%, fine-particle aggregation traces began to appear in the cross-section, and the uniformity slightly decreased (Figure 7d). The improvement in cross-sectional morphology at 1–3% CSE was microscopic evidence consistent with the enhanced interfacial compatibility mediated by DES. At appropriate CSE addition levels, the DES-mediated interfacial compatibility enabled uniform dispersion of CSE within the chitosan matrix, reducing internal voids and forming a continuous, dense microstructure [55]. This also served as the microscopic structural basis for the improved tensile strength and optimized barrier performance demonstrated in the mechanical and gas barrier property sections, respectively. The aggregation that occurred at a 5% addition level precisely corresponded to the decline in mechanical strength and barrier performance and explained the performance changes from the microscopic morphology perspective.

#### 3.4.3. FTIR

**Figure 8:**
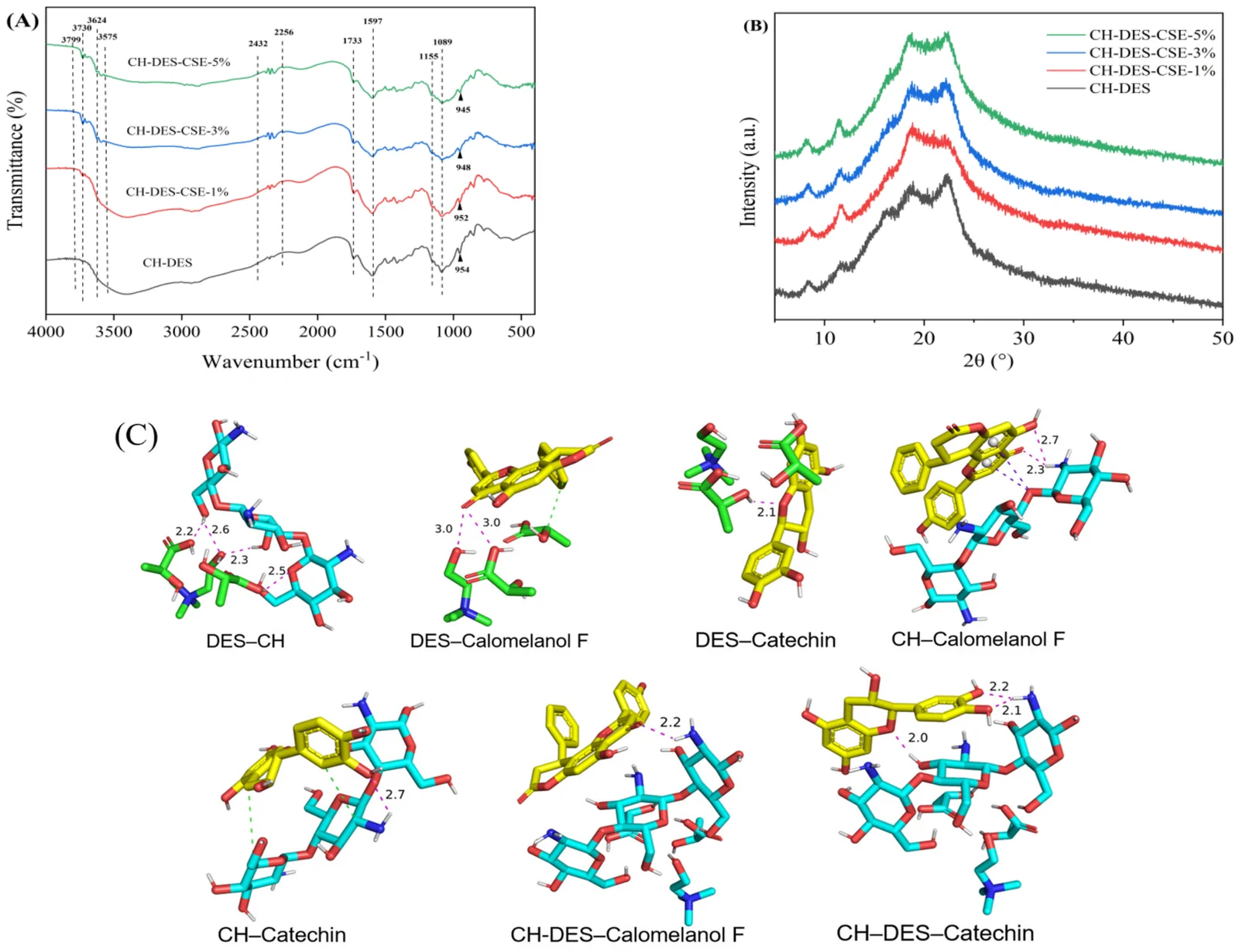
The FTIR spectra (**A**) and XRD patterns (**B**) of different films. Three-dimensional docking models (**C**) of CSE polyphenols (Calomelanol F and Catechin) with CH and DES.

Figure 8A shows the FTIR spectra of different films. As shown in Figure 8A, a broad absorption peak was observed at 3000–3600 cm^−1^, which reflects the stretching vibrations of O-H and N-H [9]. All films exhibited three absorption peaks at 1455 cm^−1^ (-CH_3_ bending), 1375 cm^−1^ (-CH bending), and 1123 cm^−1^ (-C-O stretching). These peaks have been confirmed to be characteristic of lactic acid [20]. There was also an absorption peak at 1733 cm^−1^ assigned to the C=O stretching vibration of -COOH group [25]. The same results were also obtained in the work of Yu et al. (2024) [21]. The vibration peak at 1597 cm^−1^ was stretching vibration of C=O group in the amide group. The vibration peak at 1557 cm^−1^ was N-H bending vibration in the amide group. The absorption peak at 1155 cm^−1^ was antisymmetric stretching of C-O-C bond. The C-O stretching vibrations at 1089 cm^−1^ and 1031 cm^−1^ can be explained as characteristic of chitosan structure [56]. Typical peaks of choline chloride were observed at 1483 cm^−1^ and 954 cm^−1^ [21], where the band at 954 cm^−1^ corresponds to the C–C–O vibration in the CH–DES matrix [19].

Compared with CH–DES, the CH–DES–CSE film showed new absorption peaks at 3730, 3712, 3624, and 3575 cm^−1^, which likely arise from O-H stretching vibrations introduced upon CSE addition [42]. With the increase in CSE content, the absorbance intensity of the broad band at 3575–3799 cm^−1^ gradually increased. This is attributed to the increase in the number of free -OH groups in CSE and the formation of intermolecular hydrogen bonds. Notably, this trend nearly reached a plateau when the CSE loading was 5%. This observation may be attributed to the possibility that the potential bridging-dispersion capacity of DES within the system is approaching saturation. It is difficult for excessive polyphenols to achieve stable dispersion through hydrogen bond anchoring with the chitosan matrix, and they instead undergo physical aggregation [57]. Furthermore, according to the UV-Vis full-wavelength scanning results for film color and opacity, no new characteristic absorption peaks were observed, indicating that no covalent bonds were formed between CSE and the chitosan matrix. The interaction between the two was mainly through non-covalent forces such as hydrogen bonds. This is in agreement with the multiple hydrogen bond interactions predicted by the 3.4.5 molecular docking simulation. This spectral evolution is consistent with previous reports on chitosan-based films incorporated with plant extracts [15,58]. The abundant hydroxyl groups from CSE can form extensive intermolecular hydrogen bonds with chitosan and DES components. The formation of new hydrogen bonds might constitute a plausible molecular basis for the potential bridging function of DES. Meanwhile, the reinforced hydrogen bond network also offers a molecular-level mechanism to explain the denser microstructure, enhanced mechanical performance and improved barrier properties observed in the previous characterizations.

#### 3.4.4. XRD

As shown in the XRD pattern of different films (Figure 8B), chitosan exhibited semi-crystalline properties, with characteristic diffraction peaks occurring near 11.6° and 20° [24]. In this study, the characteristic diffraction peaks were located at 11.6° and 18.7°, corresponding to the hydrated microcrystalline structure and amorphous structure of chitosan, respectively [59]. All films exhibited two diffraction peaks at approximately 8.4° and 22.2°, indicating the presence of certain crystal phases or ordered structures.

After CSE addition, the position of the two peaks at 11.6° and 18.7° shifted to the left, and the offset ranges were 11.52–11.36° and 18.64–18.48°, respectively. The largest 2θ offset distance appeared when the CSE content was 5%, and the peak intensity increased with the increase in CSE content. From these results, it can be concluded that CSE addition could enlarge the chitosan lattice and improve crystallinity [60].

Moreover, compared with CH–DES, the characteristic diffraction peak at 16.2° disappeared after CSE addition. The result may be due to the fact that hydrogen-bond formation between polyphenols and other film components may produce better lattice structure [61]. In previous published papers, it had also been reported that CSE may induce the rearrangement of chitosan molecular chains through the hydrogen bond network, further reducing the defects in the amorphous region [62]. Meanwhile, polyphenols usually behave as “biological crosslinking agents” to promote the ordered stacking of chitosan molecular chains and improve crystallinity [41]. The hydrogen bond between CSE and CH–DES may have changed the crystal structure of chitosan, leading to the disappearance of some characteristic peaks [63]. These conclusions were strongly supported by the FTIR results.

#### 3.4.5. Molecular Docking Results

Molecular docking can predict the binding conformation and binding strength between ligands and targets, thereby providing a theoretical basis for experiments [64]. To clarify the intermolecular interactions among CH, DES, and CSE, as well as their regulatory mechanism of film properties, molecular docking simulations were performed in this study. Figure 8C displays the optimal binding conformations of DES with calomelanol F and catechin, CH with calomelanol F and catechin, and the CH–DES complex with the two CSE-derived polyphenols. The pink dotted line, purple dotted line and green dotted line represent hydrogen bonds, π interactions and hydrophobic interactions (Figure 8C), respectively. The results suggest the existence of multiple intermolecular interactions among film components. Specifically, four hydrogen bonds were identified between DES and CH with an overall binding energy of −2.6 kcal/mol. One hydrogen bond was formed between DES and catechin (binding energy: −2.3 kcal/mol). In contrast, DES and calomelanol F exhibited both hydrogen bonding and hydrophobic interactions (binding energy: −2.5 kcal/mol). For CH–polyphenol systems, CH interacted with calomelanol F via π interactions and hydrogen bonds (−3.4 kcal/mol) and with catechin via hydrogen bonding and hydrophobic interactions (−3.1 kcal/mol). Notably, the CH–DES–calomelanol F and CH–DES–catechin ternary systems were stabilized by multiple hydrogen bonds, with binding energies of −3.8 kcal/mol and −3.5 kcal/mol, respectively. Consistent with previous studies, hydrogen bonds, π interactions and hydrophobic interactions are typical non-covalent weak interactions, which generally produce relatively low binding energies in molecular docking simulations [65]. The more negative binding energies of the ternary systems compared with the binary systems imply that they may possess higher structural stability. These docking results suggest that extensive hydrogen bonding interactions may exist among DES, CH and the main polyphenols in CSE (Figure 8C). In this composite system, DES is speculated to potentially act as a bridging medium and aid the solubilization of CSE components. Specifically, DES anchors the CH molecular backbone via four hydrogen bonds, which are formed between the hydroxyl hydrogen of CH and the carbonyl oxygen of DES, thereby stabilizing the CH–DES intermolecular association [55]. DES can anchor the CH backbone and the polyphenol molecules through hydrogen bonds. Consequently, with the assistance of π-interactions and hydrophobic interactions, a continuous three-dimensional hydrogen bond network is formed. These molecular docking findings provide a plausible molecular-level explanation for the improved compatibility and enhanced mechanical properties observed in our experiments. Furthermore, they are consistent with the FTIR and XRD results presented earlier. The formation of multiple hydrogen bonds revealed by docking offers a possible explanation for the new O-H stretching peaks in FTIR spectra and the increased crystallinity observed in XRD patterns. In summary, these results provide molecular level insights into potential interaction mechanisms among the components.

### 3.5. Preservation Effect of Composite Films

#### 3.5.1. Appearance of Strawberries

**Figure 9:**
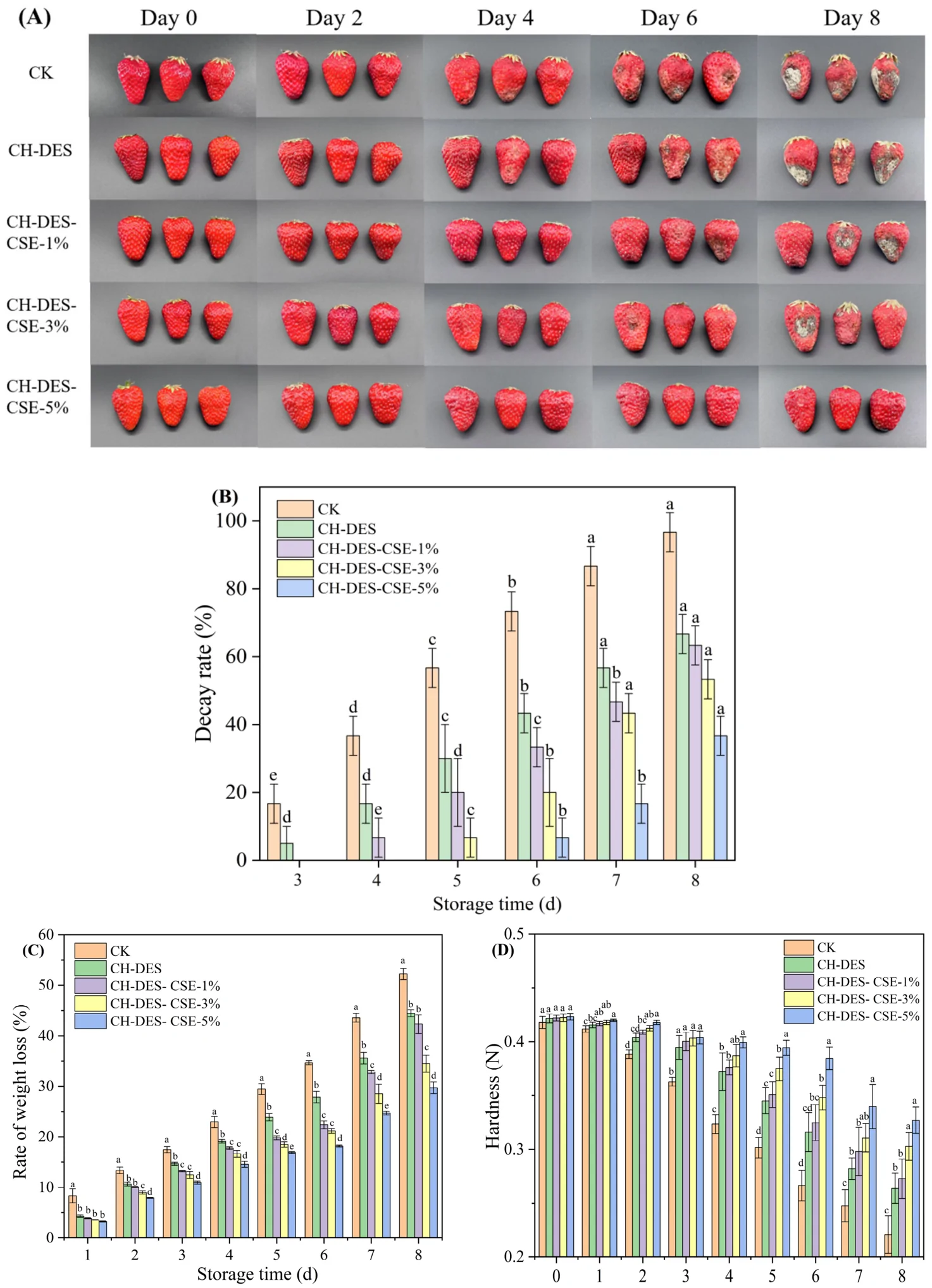

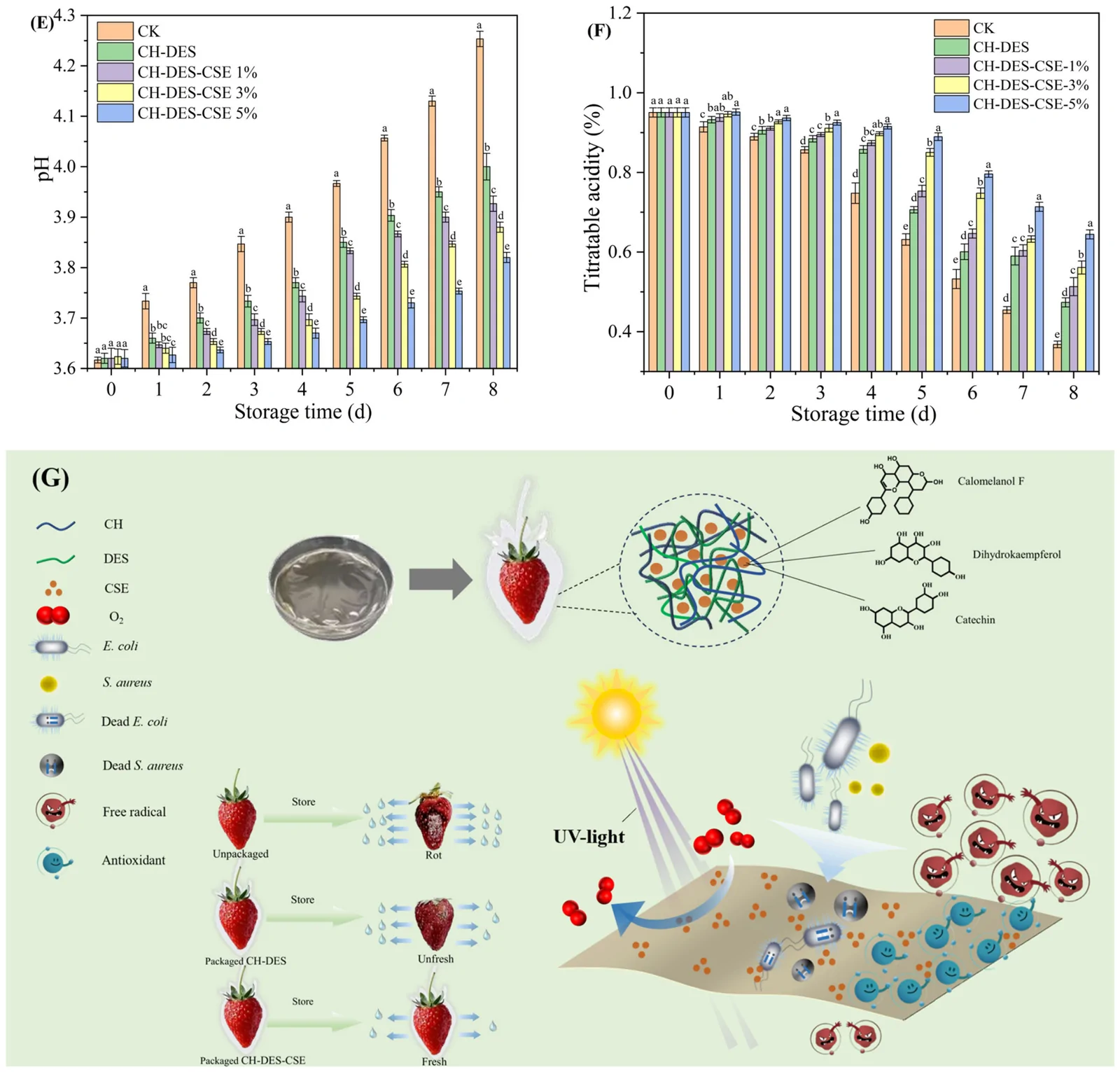
The preservation effect of films treatment on strawberries: (**A**) visual images of different treatment groups, (**B**) decay rate changes, (**C**) weight loss rate changes, (**D**) hardness changes, (**E**) pH changes, (**F**) titratable acidity changes, (**G**) possible mechanism diagram of films extending strawberry storage period (Different lowercase letters above bars indicate significant differences among treatments at the same storage time (*p* < 0.05).).

To visually evaluate the preservation performance of composite films, strawberries packaged with different films were stored at 25 °C for 8 days, and the overall changing trends of fruit appearance are summarized in Figure 9A. Generally speaking, as the storage time increases, the deterioration of strawberries becomes more severe. However, the films with CSE added show a dose-dependent antibacterial effect.

On the 4th day of storage, local decay occurred in the CK group. A small amount of damage was observed in the CH–DES group. In contrast, no obvious changes were found in the CSE-containing groups. As storage time prolonged to day 6, large-area rot spread across all fruit in the CK group. Severe decay was also observed in the CH–DES group, whereas strawberries wrapped by CH–DES–CSE-1% and CH–DES–CSE-3% films only developed obvious rot lesions until day 8. Notably, the CH–DES–CSE-5% group still maintained a good appearance on the 8th day, which was still within the acceptable range for consumers.

#### 3.5.2. Decay Rate

The quantitative decay rate data of strawberries under various packaging treatments after 8 days of storage are displayed in Figure 9B, which were consistent with the visual appearance results above. The decay rate decreased significantly with the increase in CSE addition concentration.

After 8 days of storage, the decay rate of the CK group was almost 100%. The decay rate of the CH–DES group was 66.67%. With the increase in CSE addition, the decay rate showed a significant downward trend. After 8 days of storage, the decay rates of the CH–DES–CSE-1% and CH–DES–CSE-3% groups were 63.33% and 53.33%, respectively. Moreover, the decay rate in the CH–DES–CSE-5% group was only 36.67%, which was 60.00% lower than that of the CK group. The decay process of strawberries during storage was influenced by various factors. It is proposed that the films may function as an oxygen-barrier layer, lowering O_2_ permeability, which could potentially suppress strawberry respiration and thereby delay fruit ripening. Second, the film’s superior barrier and antibacterial properties reduced oxidation and microbial decay [39].

#### 3.5.3. Physicochemical Properties of Strawberries

Weight loss rate, fruit hardness, pH value and titratable acidity (TA) are four core physicochemical indicators reflecting postharvest senescence of strawberries. Continuous respiratory metabolism of strawberries simultaneously consumes organic acids and sugars, triggering water transpiration, cell wall degradation, fruit softening, reduced TA and elevated pH. The effects of different packaging film treatments on these four indicators of strawberries are shown in Figure 9C–F.

During storage, the weight loss rate of strawberries exhibited an increasing trend (Figure 9C). After 8 days of storage, the weight loss rate of unpackaged strawberries was 52.22%. In contrast, the weight loss rate in the CH–DES–CSE-5% film group was 29.69%, which was 22.53% lower than that of the CK group. As shown in Figure 9D, the hardness of strawberries in all groups decreased with the increase in storage time. After 8 days of storage, the hardness of the CK group was 0.22 N. For the CH–DES–CSE film group, it remained at 0.30–0.33 N, which was 37.10% to 47.96% higher than that of the CK group. A plausible explanation is that the CH–DES–CSE film might mitigate strawberry respiratory metabolism and help preserve cellular structure [16]. During the storage of strawberries, the organic acids and sugars will be continuously consumed due to the respiratory metabolism. This can lead to a gradual decrease in organic acid content and an increase in pH value [66]. Figure 9E shows the pH and TA changes in strawberries during storage. As storage time extended, pH gradually increased while TA decreased. After 8 days of storage, CH–DES–CSE-5% exhibited a significantly lower pH of 3.82 than CK or CH–DES (*p* < 0.05). TA in the CK group decreased most rapidly on day 3, a response that could be associated with microbial contamination, which may accelerate strawberry respiratory metabolism [67].

Collectively, the above findings including visual appearance, decay rate and physicochemical indicators can be partly interpreted by the proposed multi-functional preservation mechanisms of CH–DES–CSE composite films, as illustrated in Figure 9G. The effectiveness of strawberry preservation was constrained by respiration, water loss, oxidative decay, and microbial decay [68]. The thin skin and high respiration rate of strawberries render them prone to rapid water loss and wilting [69]. The film used in this study has a good preservation effect on strawberries. Fresh strawberries were covered with the prepared active packaging films. This film has good water vapor barrier properties, thereby reducing water loss and improving strawberry quality. UV-induced photooxidation also compromises the quality of many fruits [69]. The films with lower UV transmission can effectively protect fruits from photooxidation [21]. The film used in the present study has significant UV blocking properties, which were also one of the reasons for extending the shelf life of strawberries (Section 3.1.1). Importantly, strawberries were susceptible to microbial contamination and rapidly decay during storage. In addition, the main components of CSE used in the film are polyphenolic compounds. These compounds can bind with electrons in free radicals, resulting in improved antioxidant activity of the film (Section 3.2.1). They can also inhibit the growth of microorganisms (Section 3.2.2). In summary, these factors are suggested to constitute the major plausible mechanisms through which CH–DES–CSE films extend the shelf-life of strawberries.

## 4. Conclusions

In this study, the active packaging films were prepared using CH, DES, and CSE. And the preservation effect on strawberries was also evaluated. DES exerted a critical plasticizing effect, endowing the films with enhanced flexibility. The addition of CSE significantly improved the antioxidant and antibacterial activities of the film solutions. Meanwhile, combined incorporation of DES and CSE contributed to improved antibacterial capacity of the composite film. The UV-vis spectroscopy results showed that as the CSE content increased, the UV blocking performance of the film significantly improved. Furthermore, SEM, FTIR, and XRD results indicated that the film structure became denser, with strong hydrogen bond interactions between CSE and the CH–DES matrix, leading to good compatibility and improved film-forming properties. Molecular docking results suggested that DES could form hydrogen bonds, which may bridge the interactions between CH and CSE polyphenols and contribute to the formation of the CH–DES–CSE ternary network. This finding provided molecular-level theoretical support for the improved film performance. Notably, film packaging reduced decay and moisture loss while maintaining firmness and acid-related quality attributes of strawberries. The decay rate of strawberries packaged using CH–DES–CSE-5% was 60% lower than that in the CK group. In conclusion, CSE can be employed as a bioactive agent in CH-based films to enhance their preservation properties.

## Figures and Tables

**Table 1:** The physicochemical properties of different films.

Samples	L*	a*	b*	ΔE	Thickness (mm)	Opacity	TS (MPa)	EB (%)	MC (%)	WS (%)
CH–DES	39.82 ± 0.29 ^a^	−0.51 ± 0.14 ^c^	1.34 ± 0.45 ^c^	59.19 ± 0.28 ^b^	0.037 ± 0.001 ^c^	2.02 ± 0.12 ^c^	12.16 ± 0.17 ^d^	85.60 ± 0.80 ^a^	30.61 ± 1.44 ^a^	25.60 ± 1.17 ^c^
CH–DES–CSE-1%	38.58 ± 0.17 ^b^	−0.31 ± 0.07 ^b^	2.21 ± 0.32 ^bc^	63.68 ± 1.32 ^ab^	0.039 ± 0.002 ^c^	2.33 ± 0.10 ^b^	17.54 ± 0.42 ^c^	72.63 ± 0.81 ^b^	17.78 ± 0.21 ^b^	30.52 ± 0.47 ^b^
CH–DES–CSE-3%	36.67 ± 0.24 ^c^	−0.08 ± 0.07 ^a^	3.30 ± 0.86 ^ab^	70.30 ± 5.45 ^a^	0.052 ± 0.001 ^b^	2.51 ± 0.08 ^b^	22.99 ± 0.81 ^a^	66.40 ± 0.56 ^c^	16.73 ± 0.28 ^bc^	31.85 ± 0.71 ^b^
CH–DES–CSE-5%	34.35 ± 0.31 ^d^	0.04 ± 0.03 ^a^	3.73 ± 0.56 ^a^	71.17 ± 5.71 ^a^	0.060 ± 0.002 ^a^	2.77 ± 0.09 ^a^	19.31 ± 0.41 ^b^	55.93 ± 1.33 ^c^	14.74 ± 0.16 ^c^	37.16 ± 2.0 ^a^

TS: tensile strength, EB: elongation at break, MC: moisture content, WS: water solubility. Values with different lowercase letters within the same column denote significant differences among different film samples (*p* < 0.05).

**Table 2:** The antibacterial activity and antibacterial zone of different film solutions.

Samples	*E. coli* (mm)	Antibacterial Activity	*S. aureus* (mm)	Antibacterial Activity
CH–DES	20.79 ± 0.28 ^d^	+++	21.74 ± 0.24 ^d^	+++
CH–DES–CSE-1%	22.18 ± 0.24 ^c^	+++	22.61 ± 0.27 ^c^	+++
CH–DES–CSE-3%	26.91 ± 0.31 ^b^	+++	24.29 ± 0.18 ^b^	+++
CH–DES–CSE-5%	27.61 ± 0.18 ^a^	+++	27.15 ± 0.20 ^a^	+++

Different lowercase letters within the same column indicate significant differences among film samples (*p* < 0.05).

## Data Availability

The original contributions presented in the study are included in the article/Supplementary Materials, further inquiries can be directed to the corresponding author.

## Supplementary Materials

The following supporting information can be downloaded at: https://www.mdpi.com/article/10.3390/foods15183217/s1, Table S1: HPLC—MS identified the chemical composition from the CSE.

## Abbreviations

The following abbreviations are used in this manuscript:

CH	Chitosan
DES	Deep eutectic solvent
CSE	Chestnut shell extract
DPPH	2,2-diphenyl-1-picrylhydrazyl
ABTS	2,2′-azino-bis(3-ethylbenzothiazoline-6-sulfonic acid)
AZD	Antibacterial zone diameter
FTIR	Fourier transform infrared spectroscopy
XRD	X-ray diffraction
HBA	Hydrogen bond acceptor
HBD	Hydrogen bond donor
ChCl	Choline chloride
WVTR	Water vapor transmission rate
*E. coli*	*Escherichia coli* SHMCCD25015
*S. aureus*	*Staphylococcus aureus* ATCC 6538
LB	Luria-Bertani
TS	Tensile strength
EB	Elongation at break
MC	Moisture content
WS	Water solubility
OTR	Oxygen transmission rate
WCA	Water contact angle
TGA	Thermogravimetric analysis
SEM	Scanning electron microscope
TA	Titratable acidity
