# Supplementary material for: Reinforced Chitosan Active Film with Deep Eutectic Solvent and Chestnut Shell Extract: Physicochemical Characteristics, Structure, and Application in Strawberry Preservation
Source: Foods. 2026 Sep 11;15(18):3217. doi: 10.3390/foods15183217 (PMC13605491; doi:10.3390/foods15183217)
Supplement: Supplementary file 1 [file foods-15-03217-s001.zip › foods-4520827-supplementary.pdf]

Reinforced chitosan active film with deep eutectic solvent and chestnut shell extract: Physicochemical characteristics, structure, and application in strawberry preservation

Jialin Xue [1, 2], Yating Sun [1, 2], Meng Wang [2], Lixiang Huai [1, 2], Yuting Xiao [1, 2], Zhenyin Lu [1, 2], Zhongxu Li [1, 2], Xianjin Zhou [3], Haoran Wang [1,2], Ruiguo Cui [1, 2], Lijun Song [2, 4, 5*]

[1] College of Food Science and Technology, Hebei Normal University of Science and Technology, Qinhuangdao 066600, China.

[2] Chestnut Research Center of Hebei Normal University of Science and Technology, Qinhuangdao 066604, China.

[3] Bazhoujiamu Agroscience Co., Ltd., Korla 841000, China.

[4] Hebei Key Laboratory of Natural Products Activity Components and Function, Hebei Normal University of Science and Technology, Qinhuangdao 066004, China.

[5] Hebei Fruit Processing Technology Innovation Center, Hebei Normal University of Science and Technology, Qinhuangdao 066600, China.

Corresponding author: Chestnut Research Center of Hebei Normal University of Science and Technology, Qinhuangdao, 066004, Hebei, China.

E-mail: slj176@163.com (Lijun Song).

1. Supplementary table

Table S1. HPLC - MS identified the chemical composition from the CSE.

Peak	RT (min)	Compound	Neutral mass (Da)	$[M-H]^-$ (m/ z)	$[M+H]^+$ (m/z)	Fragment ions (m/z)	Formula
Flavonoids							
1	20.20	Epicatechin	290	289.07129		245, 205, 179	$C_{15}H_{14}O_6$
2	20.33	Catechin 7-glucoside	452	451.12408		289, 245, 199	$C_{21}H_{24}O_{11}$
3	20.49	Dihydrokaempferol	288		289.06943	259, 165, 153, 134, 107	$C_{15}H_{12}O_6$
4	22.46	Catechin	290		291.08511	153, 137, 109	$C_{15}H_{14}O_6$
5	26.03	Manniflavanone	590		591.11127		$C_{30}H_{22}O_{13}$
6	27.82	Kanzonol I	436		437.23422	221, 68	$C_{27}H_{32}O_5$
7	27.86	Sanaganone	344		345.05728	330, 327, 317	$C_{22}H_{16}O_4$
8	28.24	Honyucitrin	406		407.18689	389, 379	$C_{25}H_{26}O_5$
9	28.33	Licoflavone B	390		391.19278	253	$C_{25}H_{26}O_4$
10	33.91	Isorhamnetin-3-O-glucoside	478		479.11667	465, 421, 317	$C_{22}H_{22}O_{12}$

11	34.59	Flemiphilippinone A	508		509.29153	439, 383, 305, 278, 249, 177	$C_{31}H_{40}O_6$
12	35.54	Calomelanol F	400		401.10416	373,357,324	$C_{24}H_{16}O_6$
Phenolic acids							
13	18.89	2-O-Galloylgalactaric acid	362		363.05896	319, 210	$C_{13}H_{14}O_{12}$
14	28.66	Sinapinic acid	224	223.06105		199, 191	$C_{11}H_{12}O_5$
15	29.42	Caffeic acid 3-O-glucuronide	356	355.06998		283, 199, 179	$C_{15}H_{16}O_{10}$
16	35.6	Glucosyringic acid	360		361.11206	199	$C_{15}H_{20}O_{10}$
17	48.88	Caffeic acid	180		181.04875	163, 145, 135, 91,89	$C_9H_8O_4$
Phenolic							
18	19.1	Procyanidin B2	578	577.13428		559, 451, 425, 407, 289	$C_{30}H_{26}O_{12}$
19	21.38	Epiafzelechin 3-O-gallate	426	425.08740		379, 299, 199	$C_{22}H_{18}O_9$
20	25.22	2,6-Bis[(2-hydroxy-5-methylphenyl) methyl]-4-methylphenol	348		349.1821		$C_{23}H_{24}O_3$

21	25.35	Procyanidin A1	576	575.11896		566, 539, 449, 407, 327, 289	$C_{30}H_{24}O_{12}$
22	26.82	Phellibaumin D	424	423.07236		299, 355, 379	$C_{22}H_{16}O_9$
23	30.45	Procyanidin A2	576	575.12006		449, 394, 287	$C_{30}H_{24}O_{12}$
24	32.05	2,6-bis [(4-hydroxy-3,5-dimethylphenyl) methyl] -4-methylphenol	376		377.21359		$C_{25}H_{28}O_3$
25	32.05	1,2-Diacetoxy-4,7,8-trihydroxy-3-(4-hydroxyphenyl) dibenzofuran	424		425.08548	382, 339, 305	$C_{22}H_{16}O_9$
26	32.86	Epigallocatechin gallate glycoside	666	665.19293		593, 521, 449, 377	$C_{27}H_{38}O_{19}$
27	33.27	Ellagic acid	302	300.99866		201, 257, 44	$C_{14}H_6O_8$
28	37.93	Cumarin derivatives	472		473.12482	401, 329, 257, 185	$C_{27}H_{20}O_8$
Esters							
29	28.56	Malicaciddimethylester	162	161.04564		89	$C_6H_{10}O_5$
30	35.54	D-Gluconic acid, methylester, 2,3,4,6-tetraacetate	378		379.12241	293, 250	$C_{15}H_{22}O_{11}$
Terpenes							
31	26.6	Glycyrrhizin	392		393.20792	349, 305	$C_{25}H_{28}O_4$
32	29.42	Asperuloside	414	413.11188		341, 269	$C_{17}H_{18}O_{12}$

33	29.68	Catalpol	362		363.12747	345, 333, 205, 203	$C_{15}H_{22}O_{10}$
34	36.82	Morroniside	406	405.14011		333, 189	$C_{17}H_{26}O_{11}$
35	37.87	Genipin 1-β-D-gentiobioside	550	549.18231		449, 377, 333, 305, 189	$C_{23}H_{34}O_{15}$
36	37.96	Asperulosidic acid	432		433.13284	415, 390, 389, 270	$C_{18}H_{24}O_{12}$
